# Detection of Quebec Polyomavirus DNA in Samples from Different Patient Groups

**DOI:** 10.3390/microorganisms9051082

**Published:** 2021-05-18

**Authors:** Carla Prezioso, Marijke Van Ghelue, Valeria Pietropaolo, Ugo Moens

**Affiliations:** 1 Department of Public Health and Infectious Diseases, “Sapienza” University of Rome, 00185 Rome, Italy; carla.prezioso@uniroma1.it; 2 IRCSS San Raffaele Pisana, Microbiology of Chronic Neuro-degenerative Pathologies, 00163 Rome, Italy; 3 Department of Medical Genetics, Division of Child and Adolescent Health, University Hospital of North Norway, 9038 Tromsø, Norway; marijke.van.ghelue@unn.no; 4 Department of Clinical Medicine, Faculty of Health Sciences, University of Tromsø—The Arctic University of Norway, 9037 Tromsø, Norway; 5 Department of Medical Biology, Faculty of Health Sciences, University of Tromsø—The Arctic University of Norway, 9037 Tromsø, Norway

**Keywords:** cerebrospinal fluid, HI-loop, HIV, multiple sclerosis, nasopharyngeal aspirates, pregnant women, systemic lupus erythematosus, VP1, urine

## Abstract

Polyomaviruses infect many species, including humans. So far, 15 polyomaviruses have been described in humans, but it remains to be established whether all of these are genuine human polyomaviruses. The most recent polyomavirus to be detected in a person is Quebec polyomavirus (QPyV), which was identified in a metagenomic analysis of a stool sample from an 85-year-old hospitalized man. We used PCR to investigate the presence of QPyV DNA in urine samples from systemic lupus erythematosus (SLE) patients (67 patients; 135 samples), multiple sclerosis patients (n = 35), HIV-positive patients (n = 66) and pregnant women (n = 65). Moreover, cerebrospinal fluid from patients with suspected neurological diseases (n = 63), nasopharyngeal aspirates from patients (n = 80) with respiratory symptoms and plasma samples from HIV-positive patients (n = 65) were examined. QPyV DNA was found in urine from 11 (16.4%), 10 (15.4%) and 5 (14.3%) SLE patients, pregnant women, and multiple sclerosis patients, respectively. No QPyV DNA could be detected in the other samples. Alignment with the only available QPyV sequence in the GenBank revealed amino acid substitutions in the HI-loop of capsid protein VP1 in 6/28 of the isolates. Our results show that QPyV viruria can occur, but whether it may cause clinical symptoms in the patients remains to be determined.

## 1. Introduction

Polyomaviruses are non-enveloped viruses with an icosahedral capsid that surrounds a circular double-stranded DNA genome of approximately 5000 base pairs [1]. Their genome contains at least four open reading frames encoding the regulatory proteins large T antigen (LT) and small t antigen, and the capsid proteins VP1 and VP2 [1]. Some polyomaviruses produce additional regulatory proteins and capsid proteins [1,2,3]. Polyomaviruses were first described in rodents and later in birds [4,5,6]. Recently, polyomaviruses have also been isolated from fish and polyomavirus sequences have also been identified in invertebrates, amphibians, and reptiles [3,7]. The first human polyomaviruses, BK polyomavirus and JC polyomavirus, were reported in 1971 in back-to-back publications [8,9]. Since then, 12 novel polyomaviruses have been described in human samples [10,11,12,13,14,15,16,17,18,19,20]. It remains to be established whether all these viruses are genuine human polyomaviruses (HPyVs), but serological studies have shown that >60% of the healthy adult population has antibodies against all of these HPyVs, justifying classifying them as HPyV [21]. Exceptions are HPyV9, HPyV12, New Jersey polyomavirus (NJPyV) and Lyon-IARC polyomavirus (LIPyV) for which the seroprevalence is ≤20% [22]. Some of these HPyVs have been shown to play a role in human diseases. BKPyV and JCPyV can cause polyomavirus-associated nephropathy in renal transplant patients. Moreover, BKPyV is also associated with hemorrhagic cystitis in hematopoietic stem cell transplants (HSCT), and a role of BKPyV in urothelial carcinomas is emerging [23,24,25]. JCPyV is the cause of progressive multifocal leukoencephalopathy [26]. Merkel cell polyomavirus (MCPyV) is an etiological factor of Merkel cell carcinoma [27,28]. HPyV6 and HPyV7 are associated with pruritic and dyskeratotic dermatoses [29,30,31,32,33], and HPyV6 also seems to be associated with Kimura disease and patients with dermatopathic lymphadenitis [34,35]. Trichodysplasia spinulonsa-associated polyomavirus (TSPyV) is the causative agent for trichodysplasia spinulosa, a proliferative dedifferentiated skin disorder [36,37]. A role for the other HPyVs in human diseases has not been established. 

In 2019, a partial polyomavirus-like sequence was originally identified in the metagenomic sequencing data of a feces sample from an 85-year-old man hospitalized in Montreal, Quebec, Canada [38]. The sequence displayed highest homology with HPyV6 and HPyV7. The complete genome of the virus was obtained by rolling circle amplification, and the authors suggested naming this new polyomavirus Quebec polyomavirus (QPyV). The QPyV genome is 80% identical to HPyV7 and 67% to HPyV6 at the nucleotide level [38]. The genoprevalence and seroprevalence of this novel polyomavirus have not been studied, and it remains to be determined whether this is truly an HPyV. This prompted us to investigate whether QPyV DNA could be detected in clinical samples of healthy individuals and different patient groups. Our results show that QPyV sequences could be detected in the urine of systemic lupus erythematosus (SLE) patients, multiple sclerosis (MS) patients and pregnant women, but not in any of the other samples tested. This suggests that QPyV viruria may not be uncommon in individuals with a compromised immune system (SLE and MS patients) or with a unique immune condition occurring during pregnancy.

## 2. Materials and Methods

### 2.1. Patients’ Samples

Archival urine samples from five anonymous SLE patients (SLE1–5) from Stavanger (Norway) were used. These samples were acquired over a 1-year period. In total, 73 samples were collected from these patients [39]. In addition, single samples from another 62 SLE patients (SLE6–67) were included. Sixty-five single archival urine specimens from healthy women who were 18–39 weeks pregnant, collected at the university hospital of Northern Norway, have been described previously [39]. Sixty-three archival cerebral spinal fluid (CSF) specimens from patients with suspected neurological complications were also tested. These samples were anonymized, and no personal or clinical data of the patients were available. The nasopharyngeal samples (n = 80) were obtained from patients with respiratory symptoms or infections and have been described previously. No further information was available for these patients [40]. The study was approved by the Regional Committees for Medical and Health Research Ethics (REK; reference number 2012/420). Thirty-five urine samples were obtained from a cohort of 35 relapsing remitting multiple sclerosis (RRMS) subjects, followed up at the Department of Human Neurosciences of Sapienza University of Rome and recruited between March 2016 and March 2018. All participants fulfilled the Italian Agency of Drug (AIFA) criteria for natalizumab treatment, and the therapeutic protocol consisted of the administration of 300 mg intravenous natalizumab every 4 weeks. The enrolled patients included 14 males and 21 females (mean age ± stand. dev.: 30.2 ± 6.6; mean months of illness: 85 ± 85.5; mean Expanded Disability Status Scale (EDSS): 1.9 ± 1.3). Urine samples were collected before natalizumab treatment (baseline: 0 infusions) and after infusions at weeks 4, 8, 12, 16 and 20. Patients signed informed consent forms based on the approval of the Ethic Committee of Policlinico Umberto I of Rome (protocol number 130/13). Sixty-six urine specimens and 65 plasma samples were collected from a cohort of 66 HIV-1-positive patients admitted to the Infectious Diseases Clinic of the Polyclinic Tor Vergata Foundation from January 2019 to December 2019. Among the enrolled patients (55 males and 11 females from 21 to 76 years old: mean age ± stand. dev.: 40.5 years old; median: 39.9 years old), 22 were new diagnoses naive to treatment and 44 were experienced patients on treatment with a triple-based antiretroviral regimen including protease/reverse transcriptase/integrase inhibitors [41]. The study was approved by the local Ethic Committee of the University Hospital Tor Vergata (Rome, Italy) (protocol number 0027234/2018, 19 December 2018), and patients’ informed consent was ascertained. 

### 2.2. DNA Purification and PCR

DNA was purified from 200 µL urine, CSF, NPA and serum samples. The samples were centrifuged for 1 min at 12,000× *g* to remove cell debris. DNA purification was performed on cell-cleared urine using the QIAamp MinElute Virus Spin Kit according to the manufacturer’s instructions (Qiagen, Hilden, Germany; cat. no. 57704). DNA was eluted in 50 µL, and 5 µL was used in PCR. To test DNA quality, APRT PCR was carried out as previously described [42].

The following primers were used to amplify a 254 bp fragment of the *VP1* gene of QPyV: 5′-CAAAGTACAACACCACTTGTAG-3′ (nucleotides in 1973-1994; GenBank accession number BK010702) and 5′- TTCTGAGGTTTCAGGAATTGCC-3′ (nucleotides 2205-2226; GenBank accession number BK010702). The PCR conditions were 40 cycles of 30 s at 90 °C, 30 s at 56 °C and 30 s at 72 °C. PCR was performed with RedTaq Ready Mix^TM^ (Sigma-Aldrich, Merck, Darmstadt, Germany; cat. no. R2523). The complete QPyV VP1 sequence was synthesized and cloned in the *Hind*III/*Xho*I sites of plasmid pcDNA3.1+C-HA by GenScript (BioPartner, Leiden, The Netherlands) and used as a positive control and to test the sensitivity of our PCR. All samples were tested in duplicate. 

### 2.3. Sequencing and Analysis of VP1

Positive PCR products were cloned by TA cloning (TOPO TA cloning kit Life Technologies, Carlsbad, CA, USA; cat. no. 45-0641) and the ligation mix was transformed in competent *E. coli* DH5 cells. Plasmid DNA was purified using the NucleoSpin Plasmid prep kit according to the manufacturer’s instructions (Machery-Nagel, Düren, Germany; cat. no. 740588.250) and sequenced with the M13 reverse primer (5′-CAGGAAACAGCTATGAC-3′) using the BigDye Terminator v3.1 Cycle Sequencing kit (ThermoFisher Scientific, Waltham, MA, USA; cat. no. 4337455) and the ABI 35000xL (Applied Biosystems, Foster City, CA, USA). The obtained sequences were compared to reference sequences deposited in GenBank (GenBank accession numbers (MZ081042-MZ081051). Sequence alignments were performed with the Clustal Omega Multiple Sequence Alignment algorithm [43].

### 2.4. Statistical Analysis

QPyV detection was summarized by counts and proportions. If continuous variables were normally distributed, they were expressed as mean ± SD; if not, they were expressed by median and range. The ꭕ^2^ test was performed to evaluate differences in the viral detection, and the Mann–Whitney U-test for non-normally distributed continuous variables was applied to analyze differences between patients. A *p* value < 0.05 was considered statistically significant.

## 3. Results

### 3.1. Detection of QPyV DNA in Specimens from Different Patient Groups

QPyV was originally identified as a sequence in a metagenomic analysis of a stool sample from an 85-year-old hospitalized man [38], but the presence of this virus in other individuals has not been studied. As the overall sequence identity at the nucleotide level between HPyV7 (HPyV6) and QPyV is 80% (67%) [38], whereas the QPyV and HPyV7 (HPyV6) VP1 are 78% (67%) identical at the nucleotide level and 87% (70%) identical at the amino acid (Figure S1), QPyV VP1-specific primers were designed with mismatch to the corresponding sequences in the HPyV6 and HPyV *VP1* gene. First, we optimized the PCR conditions. Using a serial dilution of pcDNA3-HA-QPyV-VP1 DNA, the sensitivity of our PCR under these conditions was between 0.1 and 1 fg (corresponding to between ~12 and 120 copies), but neither 10 pg HPyV6 nor 10 pg HPyV7 DNA gave an amplicon (results not shown). 

Next, we tested single urine samples from 65 pregnant women and 62 SLE patients. We also examined consecutive urine samples from five other SLE patients (patient 1: 5 samples; patient 2: 7 samples; patient 3: 13 samples; patient 4: 16 samples and patient 5: 32 samples). QPyV DNA was amplified in 10/65 (15.4%) urine specimens from pregnant women and in urine from 11/67 (16.4%) SLE patients (Table 1). Of the 62 single urine samples from SLE patients, 9 (14.5%) were QPyV positive. For the SLE patients with samples collected over time, four samples were QPyV positive; one from patient 4 and three from patient 5. QPyV DNA could be amplified from the urine of five RRMS patients (14.3%). There were no significant differences between patient groups with positive PCR results. No QPyV DNA was detected in the nasopharyngeal aspirates from individuals with symptoms or infection, or in the cerebrospinal fluid samples from patients with suspected neurological complications. All plasma and urine samples from HIV-positive patients were also negative for QPyV DNA (Table 1). 

The consecutive urine samples from SLE patients, 47 urine samples from pregnant women and 30 of the 35 urine specimens from MS patients were previously tested for HPyV6 and HPyV7 [41]. HPyV6 DNA, but not HPyV7, was detected in the QPyV DNA-positive samples SLE5.9, PW10 and PW34, but not in the samples from MS patients. This shows that simultaneous viruria of different HPyVs can occur. Co-detection of different HPyV has been previously reported, including BKPyV and JCPyV in urine from renal transplant recipients and hematological stem cell transplant patients [44,45], HPyV6 and HPyV7 in urine from pregnant women and SLE patients [41] and MCPyV and HPyV6 or MCPyV and LIPyV in the skin of liver transplant patients [46].

Our study is the first to investigate the presence of QPyV in biological samples using specific primers against the QPyV genome. QPyV was only found in urine samples from pregnant women and from SLE and MS patients. This may indicate that QPyV viruria can occur in certain patients with an aberrantly functioning immune system. However, no QPyV was detected in the urine of 66 HIV-positive patients. New viruses can be detected by the use of degenerated primers and deep sequencing techniques [47]. However, using these techniques on pooled nasal and throat swabs (community-acquired sepsis patients), muscle biopsy (pancreatic transplant patient), samples from skin (healthy individuals and HIV-positive patients), urine (renal transplant recipients, asymptomatic women and women with overactive bladder), whole blood and lung cancer tissue (lung cancer patients), plasma (febrile adults), sera (community-acquired sepsis patients), nose and mouth swabs (healthy adults and community-acquired sepsis patients), oral gargles (healthy individuals), eyebrow hairs (healthy individuals), vaginal swabs (healthy non-pregnant and pregnant women), CSF (community-acquired sepsis patients), gut (HSCT and healthy adults) and feces (healthy adults and community-acquired sepsis patients) failed to the detect QPyV, although other HPyVs were found [19,20,48,49,50,51,52,53,54,55,56,57,58]. These results suggest that QPyV is rare in humans.

### 3.2. Nucleotide Sequence Analysis

**Figure 1:**
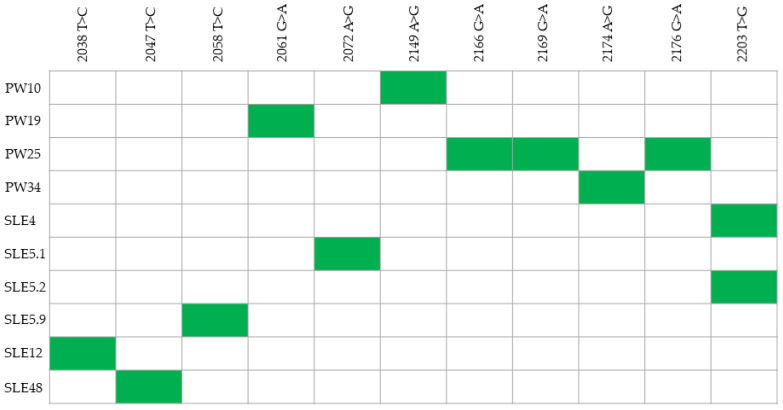
Mutations found in QPyV VP1 sequence amplified from the urine of pregnant women and systemic lupus erythematosus (SLE) patients. A green box indicates the presence of the mutation. The numbering of the nucleotides is according to the reference QPyV strain (GenBank accession number BK010702). The complete VP1 nucleotide sequence and the region sequenced in this study are shown in Figure S1.

We sequenced the PCR products and found only point mutations compared to the reference strain in some of our amplified *VP1* genes (Figure 1). None of the sequences had the same mutation, except for samples SLE4 and SLE5.2. The sequences were deposited in the GenBank (MZ081042-MZ081051).

### 3.3. Amino Acid Sequence Analysis

**Figure 2:**
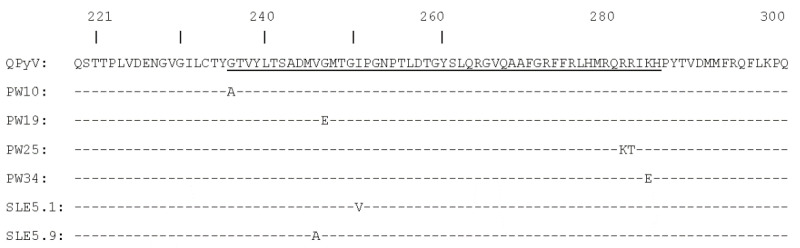
Amino acid sequence alignment of the QPyV VP1 fragment (residues 221−304). The published sequence (BK010702) is given at the top. A hyphen indicates an identical amino acid. The sequence corresponding to the HI-loop in HPyV6 and HPyV7 VP1 is underlined. Isolates with silent mutations are not shown.

Translating the nucleotide sequence into amino acids revealed that some of the mutations resulted in amino acid substitutions (Figure 2). The transition from T to C in SLE 4 and SLE5.2 changes the TTT codon specifying F298 in VP1 into the synonyms codon TTC. The silent mutation in SLE 12 changes TAT (Y) into TAC (Y), while the silent mutation of codon TCT into TCC in SLE48 leaves the S245 unaffected. All mutations were located in the region that corresponds to the HI-loop in HPyV6 and HPyV7 [59]. Every amino acid substitution was unique for a specific isolate.

We determined the sequence of residues 221–304 of the QPyV VP1, a 388 amino acid long protein (Figure S1). This fragment includes the HI-loop, which participates in the recognition of sialylated glycan receptors in all sialic-binding polyomaviruses whose structures have been determined to date [59]. However, compared to other HPyVs, this loop is extended in HPyV6 and HPyV7. The amino acid sequence of the QPyV HI-loop is 71% identical to that of HPyV6 and 76% identical to that of HPyV7. This suggests that the HI-loop of QPyV VP1 is also extended. This distinctive structure of the HPyV6 and HPyV7 HI-loop, and especially residues Y257 in HPyV6 (accession number HM011558) and L254 in HPyV7 (accession number NC_014407), appears to hinder the binding of sialic acids to the HPyV6 and HPyV7 capsids [59]. The corresponding residue in QPyV VP1 is I254. All the amino acid substitutions that we found in the QPyV of our samples reside in the HI-loop, but future research is needed to investigate the biological consequences of these mutations.

In conclusion, the genoprevalence of QPyV seems to be low, but QPyV viruria can occur in conditions of reduced immune responses (pregnancy) or in individuals with a malfunctioning immune system (SLE and MS patients). Classification of QPyV as a bona fide human polyomavirus requires additional genoprevalence and seroprevalence studies.

## Figures and Tables

**Table 1:** Prevalence of QPyV DNA in clinical samples from different patient groups.

Patient Group (n)	Specimen	QPyV Positive
SLE * patients (5) patient 1 (5 samples) patient 2 (7 samples) patient 3 (13 samples) patient 4 (16 samples) patient 5 (32 samples)	consecutive urine	0 (0%) 0 (0%) 0 (0%) 1 (6.3%) 3 (9.2%)
SLE patients (62)	urine	9 (14.5%)
pregnant women (65)	urine	10 (15.4%)
suspected neurological complications (63)	CSF	0 (0%)
respiratory symptoms or infection (80)	NPA	0 (0%)
HIV+ (66)	urine	0 (0%)
HIV+ (65)	plasma	0 (0%)
mutiple sclerosis (35)	urine	5 (14.3%)

* abbreviations: CSF = cerebrospinal fluid; NPA = nasopharyngeal aspirates; SLE = systemic lupus erythematosus.

## Data Availability

The data is contained within the article and Supplementary Materials.

## Supplementary Materials

The following are available online at https://www.mdpi.com/article/10.3390/microorganisms9051082/s1: Figure S1: Nucleotide sequence and amino acid sequence alignment of the QPyV, HPyV6, and HPyV7 VP1.
