# Supplementary material for: Detection of Quebec Polyomavirus DNA in Samples from Different Patient Groups
Source: Microorganisms. 2021 May 18;9(5):1082. doi: 10.3390/microorganisms9051082 (PMC8158138; doi:10.3390/microorganisms9051082)
Supplement: Supplementary file 1 [file microorganisms-09-01082-s001.zip › microorganisms-1227451-supplementary.pdf]

Supplementary Materials

(A)

```
HPyV6      atgccttgtcacagaaaaggaaatggaccaattcaaaagcttcccagagtaataaaaaaa      60
QPyV       atgtcttgtacaagaaaaggaaatggaccccctcagaagctcccccgagtcataagaaaa      60
HPyV7      atgccctgtcaaagaaaaggaaatggacccacacagaaacttcccagagtgataagaaaa      60
           *** * ***   *****************    ** ** ** *** **** **** ****

HPyV6      ggaggagtggaggttatggaaacagtgccactttctgaagacacaatttataaagtggag      120
QPyV       ggaggcgttgaagtcttggaaactgtgccactaacagaggacacaatgtacaaagttgaa      120
HPyV7      ggaggcgttgaagttttggatacagtaccactcactgaggaaacacagtacaaagttgaa      120
           ***** ** ** **  **** ** ** *****  * ** ** ***   ** ***** **

HPyV6      gcaattctgctgcctaattttgcttctggatccaatactgcagtataccagtctcgggga      180
QPyV       gcagttctgctgccaaactttgggaaaggacctacaagtggcctctatcaatctagaggc      180
HPyV7      gcagtgttgctgccaaactttggaaaagcagctacaactgggaactttcaatctagaggc      180
           *** *  ******* ** ****     * * * *  * **     *  ** *** * **

HPyV6      gcccccctacacctttaccgacactttggatgctggttcatctctgtgttataccctggct      240
QPyV       caggcctatcctttgacagacactttgggggcaggggctgctttgtgtttttctattgct      240
HPyV7      ctaccttatcctatgagtgacactctggggccgggggctgctttgtgttactctgttgct      240
               * **  *  * *  ****** ***   * **  *  ** ******   *  * ***

HPyV6      gttgttaatttgcctgaaattcctgaggccctgtgtgatgatactttgttggtttgggaa      300
QPyV       gctataaatttgcctgagattcctgatgctatgtgtgaagacacaatgctagtttgggaa      300
HPyV7      gttattaatttgcctgaaattcctgatgccatgtgtgaggacacaatgattgtttgggag      300
           * * * *********** ******** **  ******* ** **  ** * ********

HPyV6      gccttcagagttgaaacagaactaatatttaccccacaagtgggaagtgctggatatata      360
QPyV       gccttcaggctggaaacagatgttatatttgctccacaactgggtagtgctggataccaa      360
HPyV7      gcctataggctggaaacagaacttctatttgctccacaaatggctagttctggttatcaa      360
           ****  **  * ********  *  ***** * ****** ***  *** **** **   *

HPyV6      agagcacaaggaacccctgctggtgtagaaggttcccaaatgtacttttgggcctgtgga      420
QPyV       agatcccagggcactccagcaggaatagaaggtacccaattctacttttgggcctgtgga      420
HPyV7      agggctaatggcactctagctggaacagagggcagtcaactatacttctgggcctgtgga      420
           **  *  * ** ** *  ** **   *** **    *** * ***** ************

HPyV6      ggcagccccttggatgtcattggtattaacccagatccagaaagaatgaatgtagcagca      480
QPyV       ggaggtccccttgatgttataggaattaatccttaccctacagggtttaaagtggctgct      480
HPyV7      ggaggccccttggatgtaattggaattaaccctgaccctgaaaggctaaaggtcaatgaa      480
           **  * *** * ***** ** ** ***** **  * **   * *  * ** **    *

HPyV6      ggcctggaagggcctagtaaagagaaccaaccatctgttgcaggcattaaagctaccaga      540
QPyV       gagcttgaagtgccaggcac---------tacagatgttgcaagcctccaagcactgaga      531
HPyV7      gccctagaaggtccaggcaa---------tactgatgtagctagcctgcaagccctcaga      531
           *  ** ****  **  * *            *   *** **  ** *  ****    ***

HPyV6      aaacaagttacagctgccaattttcccattgaaatttggagtgctgatcctaccaggaat      600
QPyV       aagcaagtcaatgctgcaaactacccagtggaggtgtgggttccagatcctacaaaaaat      591
HPyV7      aagcaagtaaatgctgcaaatttcccagtagagctctgggttgcagatcccacaaaaaat      591
           ** ***** *  ***** ** *  **  * **  * ***  * * ***** ** *  ***

HPyV6      gagaactgtagatattttggcagaattgttggtggtagtgttacccctccagtggtttcc      660
QPyV       gaaaactgcagatattttggaagagtggtaggagggcaggtcacccctcctgttgtgtct      651
HPyV7      gataataccagatactttggcagagtggttggagggggagtaacccctccagttgtgtcc      651
           ** **    ***** ***** *** * ** ** **    ** ******** ** ** **

HPyV6      tttggaaatcagagcaccactccattggttgatgaaaatggagtaggtatactctgtttg      720
QPyV       tatggtaatcaaagtacaacaccacttgtagatgaaaatggtgtagggattttgtgtacc      711
HPyV7      tatgggaatcagagtaccactccccttattgatgaaaatggagttggaattttgtgtact      711
           * *** ***** ** ** ** **  *  * *********** ** ** **  * ***

HPyV6      tttggagctatatacctaacatcagcagatatgttggggatggtagggtatgctggtaac      780
QPyV       tatggcactgtgtatcttacctctgcagacatggttggaatgactggaattccaggcaat      771
HPyV7      tttggcagtgtgtatcttaccagtgctgatatggttggaatgacaggccttccaggcctt      771
           * ***   * * ** ** **    ** ** *** * ** ***   **   * * **
```

```
HPyV6    cccactctcagtgatgcctatagccagcaaagaagtgtgcaggctgcctttggaagattt    840
QPyV     cctacccttgatactggctacagtcttcagagaggggtgcaggctgcttttggcaggttt    831
HPyV7    cccactctgagtgctgactacagtaaccagagaacagtacaggctggttatggcagattt    831
         ** ** **   *  ** *** **    ** ***   ** *******  * *** ** ***

HPyV6    tttagagttcacttcagacagaggagagttaaacatccatatactgtggatatgatgttt    900
QPyV     tttagactgcatatgagacagagaagaattaagcacccatatacagtggacatgatgttt    891
HPyV7    tttagagtccactgcagacaaagaagaattaagcacccctacacagtggatatgatgttt    891
         ****** * **    ***** ** *** **** ** ** ** ** ***** *********

HPyV6    agacaattcctgcagcctcagaagccacaggtgcaagggacacaacctaatgctgtccaa    960
QPyV     aggcaattcctgaaacctcagaagcctcacattcaggggcagcaagctggtgttgtgcag    951
HPyV7    agacagtttctgcagccccagaagccacaggtgcaggggcagcaacctgctgcagtgcaa    951
         ** ** ** *** * ** ******** **  * ** ***   *** **  **  ** **

HPyV6    gaagtagtaatggaacaaatgcagccaagcatacttccaactactctggagggtgctatt    1020
QPyV     gaagttataatggagcaaatgcagcctcctaccatgcccccaactattgagggaggtcta    1011
HPyV7    gaagttacaatggagcaaatgcagcctgctactatccctccaactgttgagggaggcctt    1011
         *****   ****** ***********    *   * **  * *** * ***** *   *

HPyV6    gggtactcacccagtacaaagtttattctccaaaatggggaacttatatacccttctagt    1080
QPyV     ggatttgccccaagtagtaggtttgtgctacaaaatggggagcttatctatccaacagtc    1071
HPyV7    ggctttgctcccaccagtaagtttctgatccaaaatggagagcttatttatcccagcagc    1071
         ** *   * ** *  *  * **** *  * ******** ** ***** ** **

HPyV6    acagtagcagcaggtgctgcaaatttgtttgggcctccagtagagaaacagacaagcaag    1140
QPyV     ccctcaactgcagatgctgcaaaggtgactgtagccccacctaaaaaaacaatagggccc    1131
HPyV7    aatgctgctgcagcagctgcaaaaatatctgtagccccaaagaaaaacacagacaacaag    1131
                * ****  ********  *   **   * ***    * **

HPyV6    gaacc-tagt--aagggtga---------gctgtaa    1164
QPyV     aaagaccaaaataagaaagacaagaatgatttgtaa    1167
HPyV7    aaggaattgtaa------------------------    1143
          *
```

(**B**)

```
HPyV6    MPCHRKGNGPIQKLPRVIKKGGVEVMETVPLSEDTIYKVEAILLPNFASGSNTAVYQSRG    60
QPyV     MSCTRKGNGPPQKLPRVIRKGGVEVLETVPLTEDTMYKVEAVLLPNFGKGPTSGLYQSRG    60
HPyV7    MPCQRKGNGPTQKLPRVIRKGGVEVLDTVPLTEETQYKVEAVLLPNFGKAATTGNFQSRG    60
         * * ****** *******:******::****:*:* *****:*****... .:. :****

HPyV6    APYTFTDTLDAGSSLCYTLAVVNLPEIPEALCDDTLLVWEAFRVETELIFTPQVGSAGYI    120
QPyV     QAYPLTDTLGAGAALCFSIAAINLPEIPDAMCEDTMLVWEAFRLETDVIFAPQLGSAGYQ    120
HPyV7    LPYPMSDTLGPGAALCYSVAVINLPEIPDAMCEDTMIVWEAYRLETELLFAPQMASSGYQ    120
           * ::***. *::**:::*.:******:*:*:**::****:*:**:::*:**:.*:**

HPyV6    RAQGTPAGVEGSQMYFWACGGSPLDVIGINPDPERMNVAAGLEGPSKENQPSVAGIKATR    180
QPyV     RSQGTPAGIEGTQFYFWACGGGPLDVIGINPYPTGFKVAAELEVPGTTD---VASLQALR    177
HPyV7    RANGTLAGTEGSQLYFWACGGGPLDVIGINPDPERLKVNEALEGPGNTD---VASLQALR    177
         *::** ** **:*:*******.********* *  ::*   ** *.. :   **.::* *

HPyV6    KQVTAANFPIEIWSADPTRNENCRYFGRIVGGSVTPPVVSFGNQSTTPLVDENGVGILCL    240
QPyV     KQVNAANYPVEVWVPDPTKNENCRYFGRVVGGQVTPPVVSYGNQSTTPLVDENGVGILCT    237
HPyV7    KQVNAANFPVELWVADPTKNDNTRYFGRVVGGGVTPPVVSYGNQSTTPLIDENGVGILCT    237
         ***.***:*:*:*  ***:*:* *****:*** *******:********:*********

HPyV6    FGAIYLTSADMLGMVGYAGNPTLSDAYSQQRSVQAAFGRFFRVHFRQRRVKHPYTVDMMF    300
QPyV     YGTVYLTSADMVGMTGIPGNPTLDTGYSLQRGVQAAFGRFFRLHMRQRRIKHPYTVDMMF    297
HPyV7    FGSVYLTSADMVGMTGLPGLPTLSADYSNQRTVQAGYGRFFRVHCRQRRIKHPYTVDMMF    297
         :*::*******:**.*  * ***.  ** ** ***.:*****:* ****:**********

HPyV6    RQFLQPQKPQVQGTQPNAVQEVVMEQMQPSILPTTLEGAIGYSPSTKFILQNGELIYPSS    360
QPyV     RQFLKPQKPHIQGQQAGVVQEVIMEQMQPPTMPPTIEGGLGFAPSSRFVLQNGELIYPTV    357
HPyV7    RQFLQPQKPQVQGQQPAAVQEVTMEQMQPATIPPTVEGGLGFAPTSKFLIQNGELIYPSS    357
         ****:****::** *  .**** ******  :* *:**.:*::*:::*::********:
```

```
HPyV6       TVAAGAANLFGPPVEKQTS-KEPSKG---EL 387
QPyV        PSTADAAKVTVAPPKKTIGPKDQNKKDKNDL 388
HPyV7       NAAAAAAKISVAPKKNTDNKKEL-------- 380
              :* **::    * ::   . *:
```

Figure S1. Nucleotide sequence and amino acid sequence alignment of the QPyV, HPyV6, and HPyV7 VP1. (**A**) Nucleotide sequence alignment of the VP1 gene. The primer sequences are underlined. (**B**) Amino acid sequence alignment of the VP1 gene. The residues corresponding to the amplified gene fragment are underlined.
